# Injuries associated with housing conditions in Europe: a burden of disease study based on 2004 injury data

**DOI:** 10.1186/1476-069X-10-98

**Published:** 2011-11-10

**Authors:** Michael D Keall, David Ormandy, Michael G Baker

**Affiliations:** 1 He Kainga Oranga/Housing and Health Research Programme, University of Otago, PO Box 7343, Wellington South, New Zealand; 2 WHO Collaborating Centre for Housing Standards and Health, School of Health and Social Studies, University of Warwick, Coventry CV4 7AL, UK

**Keywords:** Injury burden, housing injury hazards, attributable risk, Europe

## Abstract

**Background:**

The authors recently undertook a study for the World Health Organization estimating the European burden of injuries that can be attributed to remediable structural hazards in the home. Such estimates are essential for motivating injury prevention efforts as they quantify potential health gains, in terms of injuries prevented, via specific environmental interventions.

**Methods:**

We combined exposure estimates from existing surveys and scenarios with estimates of the exposure-risk relationship obtained from a structured review of the literature on injury in the home and housing conditions. The resulting attributable fractions were applied to burden of injury data for the WHO European Region.

**Results:**

This analysis estimated that two specific hazards, lack of window guards at second level and higher, and lack of domestic smoke detectors resulted in an estimated 7,500 deaths and 200,000 disability adjusted life years (DALYs) per year. In estimating the environmental burden of injury associated with housing, important deficiencies in injury surveillance data and related limitations in studies of injury risk attributable to the home environment were apparent. The ability to attribute proportions of the home injury burden to features of the home were correspondingly limited, leading to probable severe underestimates of the burden.

**Conclusions:**

The burden of injury from modifiable home injury exposures is substantial. Estimating this burden in a comprehensive and accurate manner requires improvements to the scope of injury surveillance data and the evidence base regarding the effectiveness of interventions.

## Background

Home injuries present an important health burden worldwide and the home is a setting that is at least as significant as the road for injury [1]. In Europe, almost 110,000 people die each year as a result of a home/leisure injury and an estimated 32,000,000 are hospitalised [2]. The 2003-2005 home/leisure fatal injury rate is 22 per 100,000 people Europe-wide, which is more than twice the rate of road fatalities (10 per 100,000), and varies between a minimum of 12 per 100,000 in Ireland to a maximum of 72 per 100,000 in Latvia and Estonia [2]. The injury burden is particularly important for children: in Europe, home injury deaths are highest in children under 5 years of age and then sharply decrease, in contrast to road traffic deaths, which increase with age [3].

The Environmental Burden of Disease (EBD) is "the portion of the burden of disease that can be attributed to the harmful effects (i.e., the risk factors) of the surrounding environment" [4]. A recent publication by the Kuratorium für Verkehrssicherheit [2] considered that almost half of injuries in the European Union (more than 100,000 injuries annually) could be prevented given changes in exposures.

Burden of injury estimates are vitally important for motivating and orientating injury prevention efforts. They provide a crude basis for allocating resources as prevention efforts can be highly effective in some areas of minor disease burden, even though other areas attract attention because of the sheer size of the burden. A more rational basis is provided when burden of injury estimates are considered alongside estimates of the cost and effectiveness of injury prevention approaches, such as is undertaken in the development of some road safety strategies [5]. Together, such estimates can then be used to set priorities and targets that in turn guide expenditure and effort in injury prevention measures.

The large injury burden associated with the home environment merits close study of exposure to injury hazards in the home. Over time, the application of building science has led to improvements in the design of housing features, such as ergonomic studies of stair design, with likely positive implications for safety that are difficult to quantify [6]. Recent reviews of studies of the safety effects of housing improvements [7-9] have identified a few discrete areas where sufficient evidence exists to estimate the burden of injury associated with the home.

To produce an estimate of the risk of injury in the home due to housing conditions, it is necessary to focus on studies that have investigated this risk and provided a quantified relative risk estimate. One recent review by Jacobs and Baeder [7] evaluated evidence of the effectiveness of particular home injury prevention interventions. There were few home safety measures for which an associated risk ratio or odds ratio was estimated, including: the provision of window guards [10]; installation of smoke detectors [11]; provision of fencing for swimming pools [12]; legislation to limit domestic hot water temperature settings [13]; air conditioning during heat waves [14]. There is also likely to be an injury burden attributable to many other home injury hazards, (such as inadequate handrails for steps, poor lighting, slippery surfaces, poor ergonomics), but statistically significant associations between such hazards and injury occurrence have only been shown in one observational study, to our knowledge [15].

As part of the WHO burden of disease study [16], we used data on the burden of home injury in the WHO European Region to estimate the impact of modifiable features of housing on injury incidence, deaths, and Disability Adjusted Life Years (DALYs). The range of housing conditions considered was severely limited by the exposure data that were available and by gaps in the literature on the exposure-response relationship for many exposures. Consequently, our analysis was restricted to only two injury-hazard combinations: for children (aged<15 years), deaths and DALYs from falls from second level or higher windows without window guards; and for all age groups, deaths and DALYs due to domestic fires associated with lack of smoke detectors. The methods, results and limitations of current home injury burden calculations are discussed in this paper.

## Methods

Our analysis used a standard method for estimating an EBD [17]. The Population Attributable Fraction (PAF) is the proportion of disease that can be ascribed to a specified risk factor. In this context, PAF represents the proportion of injury in a population that would be prevented if exposure to remediable housing injury hazards were removed from the entire population. In this analysis we have used the following univariate formula for calculating PAF:

$$PAF=\frac{p\left(RR-1\right)}{p\left(RR-1\right)+1}$$

Where p = proportion of the population exposed, and RR is the relative risk for the condition in those exposed.

The PAF is then applied to the total burden of home injury in the WHO European Region, to estimate the proportion of cases, deaths and DALYs that is associated with specific inadequate housing conditions.

Estimating this EBD relies on the following three types of data:

• Exposure assessment - From existing estimates and scenarios. Where housing exposures cannot be estimated, we use a scenario-based approach, as outlined in the WHO report "Methodology for assessment of Environmental burden of disease" [17].

• Exposure-risk relationship - Obtained from a structured review of the literature on injury in the home and housing conditions.

• Total burden of disease - Obtained from previous WHO global burden of disease estimates based on injury reporting by states in the WHO European Region as well as the European Injury Database [18], which contains detailed injury data reported from a small sample of hospitals.

### Exposure Assessment

This component of the burden of disease calculation is an assessment of exposures to particular, potentially modifiable, housing conditions that are implicated in home injury.

#### Method for measuring children's exposure to home fall hazards

Five European countries (Greece, Norway, Poland, Scotland and Sweden) are reported to have a national law requiring guards to prevent children from falling out of windows of height more than one storey/level [19]. Apart from the existence of laws, there is little or no basis on which to assess children's exposure to such fall hazards as no representative surveys appear to be conducted at this time. Instead, we have used hospitalization and WHO data to determine exposures.

#### Methods for measuring exposure to lack of smoke detectors

England has information on the prevalence of domestic smoke detectors from its regular House Condition Survey [20]. The 2002 results estimated that 23.5% of dwellings had no smoke detectors; this improved to 15.8% in 2006. Estimates for continental Europe are available from the LARES (Large Analysis and Review of European housing and health Status) study, which was conducted in 2002/2003 in eight cities [21]. The city with the lowest rate of dwellings without smoke detectors was Bonn (75.9%), followed by Geneva (85.4%), with the poorest rate surveyed in Ferreira do Alentejo in Portugal (98.3%).

There are also some estimates available from the a web site providing estimates of domestic smoke detector prevalence in other countries [22]. Although the sources of the estimates are not always specified, the countries listed (with proportion of households without smoke detectors) include: Germany (69%), Sweden (30%), Norway (2%) and the Netherlands (32%). A population weighted average of all these estimates, using the estimates in the previous sentence plus the 2006 UK estimate and the LARES city estimates to represent other respective countries, is 66%. This will be an optimistic estimate of European prevalence of homes without smoke detectors as the larger countries represented here are relatively wealthy and are likely to have higher smoke detector fitment rates than most of the smaller European countries.

### Total burden of disease from certain types of injury in the home

The final component of the burden of disease calculation is data on the burden of disease, obtained from counts of injuries. This section describes the data sources.

For this project, two main data sources were used to estimate injury rates and totals:

1. The European Injury Database (IDB) is hosted by the European Commission and reports summarised data from a sample of hospitals in certain European countries [18]. The injury counts combine routine causes of death statistics, hospital discharge registers and data sources specific to injury areas, including road accidents and accidents at work. An estimate of population rates is also extrapolated in the web-based tables on the assumption that the sampled hospitals represent all hospitals in the country. The number of reporting hospitals is quite small and only a few countries are represented at this stage. Given these limitations, we used the IDB data only to estimate proportions of injuries of particular types in particular settings. These estimated proportions are then applied to the Global Burden of Disease data described below.

2. DALYs for injury and counts of injury deaths have also been calculated for all countries of the WHO European Region as part of the WHO GLobal Burden of Disease (GBD) Project: 2004 updatehttp://www.who.int/healthinfo/bod/en/index.html. The burden of disease due to injury is presented in the appendix according to two classes of injury: fires; child falls. These data are combined with estimates of proportions of injuries per setting derived from the IDB, described above, to estimate injury burden associated with the home setting.

The GBD data in the Appendix only lists total deaths and DALYs without classifying by the setting. However, we estimated that deaths from domestic fires constituted 90% and 82% of all fire deaths for Germany and the UK respectively by comparing the GBD data with total deaths from domestic fires [23,24]. The lower proportion in the UK could be related to the higher prevalence of domestic fire detectors in the UK. We used the UK figure of 82% as the multiplier to estimate domestic fire deaths and DALYs. This is likely to be conservative on average as the UK has one of the highest smoke detector fitment rates.

Table 1 uses data from the IDB project, showing the proportion of all home falls for children that were classified as "fall/jump from greater height" and excluded falls on the level and falls on stairs and falls of less than one metre (counts of which were all specified elsewhere in the database). These were falls occurring at the residence, but excluded falls in the yard (i.e., excluding those settings specified as: playground in residential area, garden, private driveway, parking area, garage, carport, path, walking area). By excluding falls from trees, etc, it is likely that mainly falls from windows were therefore counted. Falls from a greater height will include some falls from windows and some from balconies. A study of children aged under 15 injured in falls from heights associated with buildings in Dallas, Texas, found that 52% had fallen from windows and 45% from balconies [25]. Falls from bunk beds are unlikely to constitute a substantial portion of the injury burden of falls [26] and can obviously be prevented by not having such beds in the first place [27]. Although such studies indicate that housing features such as bunk beds and widely-spaced balcony rails could be associated with preventable injury (and hence part of the burden of injury associated with housing conditions), we will conservatively restrict the estimation of the burden only to those features that have been the focus of an intervention study. Of the various housing features related to falls, only the installation of window guards have been found to be effective to our knowledge [10].

**Table 1:** 2002-2005 average annual number of home injuries classified as "Fall/jump from greater height" (greater than 1 metre) for children aged 0-14: count of cases and proportion of all home fall injuries in the database (Source IDB).

	Cases (sample)	Incidence Rate per thousand	Rate as proportion of all fall rate
Austria	284	3.25	5.9%

Denmark	591	1	1.5%

France	2528	7	6.5%

Netherlands	<5	-	-

Portugal	<5	-	-

Sweden	278	1	2.2%

UK*	<5	-	-

*2002 only. When there are fewer than five cases in the given country per year, no figures are reported.

These IDB data show that hospitalised falls in the home setting classified as "fall/jump from greater height" were likely to constitute between 1.5% and 6.5% of all hospitalised falls in all settings for children. The rate of such injuries as a proportion of all fall injuries will vary according to factors such as children's exposure to unlatched/unguarded windows in each country concerned. The fraction of such falls as a proportion of all falls for children varies considerably between the countries shown in Table 1. It is therefore appropriate to estimate burden of disease due to children's falls from unguarded windows using a range of rates to give a realistic EBD range. The range of home fall rates applied to all the European countries is 1.5% to 6.5%. These rates are then halved to conservatively estimate rates of falls from windowsat home as a proportion of all falls on the basis of the single study we could find that analysed such injuries [25].

## Results

### Lack of smoke detectors

The exposure values for different countries are shown above. For those without exposure estimates, the population weighted average for those countries with proportions of homes without smoke detectors estimates supplied was used, a value of 66%. The population attributable fraction (PAF) for lack of smoke detectors on fire-related deaths for these countries is:

$$PAF=\frac{p\left(RR-1\right)}{p\left(RR-1\right)+1}=\frac{0.66\;\;\times \;\left(2.0-1.0\right)}{0.66\;\times \left(2.0-1.0\right)+1.0}=0.40=40\%$$

Where *p *= proportion of the population exposed, and RR is the relative risk for the condition in those exposed.

The EBD assessment for the contribution of housing conditions to fire injury deaths in the WHO European Region for those countries without exposure estimates is therefore:

Fire-related deaths attributable to lack of smoke detectors (assuming 44% fitment)

= PAF × death rate

= PAF × rate of fire-related deaths × proportion of deaths estimated to be in the home setting

= 0.40 × 37.2 × 0.82 = 12.18 fire-related deaths/1,000,000

These estimates are shown in Table 2 which also includes estimates for deaths and DALYs. As discussed above, 82% of all fire deaths are conservatively estimated to occur in the home.

**Table 2:** EBD of fire-related injury from housing conditions

	% houses	PAF	DALYs	deaths	population	DALY rate	death rate
**Country**	**without detectors**		**000s**		**000s**	**per million**	**per million**

France**	88.8%	0.47	3.97	195.30	60591.14	65.53	3.22

Germany	69.0%	0.41	2.84	162.77	82642.62	34.35	1.97

Hungary**	91.4%	0.48	1.58	70.18	10113.27	156.36	6.94

Italy**	95.8%	0.49	1.89	114.60	58433.92	32.29	1.96

Lithuania**	95.5%	0.49	1.35	58.63	3440.16	392.10	17.04

Netherlands	32.0%	0.24	0.20	8.65	16263.54	12.06	0.53

Norway	2.0%	0.02	0.02	0.94	4608.55	3.99	0.20

Portugal**	98.3%	0.50	0.68	45.48	10471.59	64.71	4.34

Slovakia**	91.2%	0.48	0.89	21.92	5386.70	165.64	4.07

Sweden	30.0%	0.23	0.30	21.60	8997.69	33.07	2.40

Switzerland**	85.4%	0.46	0.25	13.66	7392.07	34.09	1.85

United Kingdom	15.8%	0.14	0.89	51.03	60050.59	14.74	0.85

Countries without data	66.0%*	0.40	182.72	6757.80	554885.22	329.29	12.18

**Grand Total**			**197.57**	**7522.55**	**883277.04**	**223.67**	**8.52**

*a weighted European average (see text)

**These estimates are from the LARES survey, which only surveyed one city within the respective country

Based on estimates shown in the second column of Table 2 of exposure to housing without smoke detectors and reported fire-related DALYs and deaths, this EBD represents 7,500 additional deaths and almost 200,000 DALYs across the WHO European Region that can be attributed to lack of smoke detectors (Table 2).

### Lack of window guards

Table 3 shows two contrasting scenarios of children's exposure to housing without window guards on windows higher than ground floor level and reported fall-related DALYs and deaths. These estimates are combined with high and low scenarios of the proportion of child-hospitalised falls that were from higher windows (from Table 1). This EBD represents between 200-1,300 additional deaths across the WHO European Region that can be attributed to lack of window guards.

**Table 3:** EBD of child fall injury from lack of window guards.

High or low impact	Proportion of falls that are	Scenario	PAF	DALYs	deaths	pop	DALY rate	death rate
scenario	from home windows	% with guards		000s		000s	per million	per million
high	3.25%	10%	47.4%	5.7	18.3	162,652	35	0.11

low	0.75%	50%	33.3%	0.9	3.0	162,652	6	0.02

Table 3 uses two exposure scenarios across the WHO European Region: high impact scenario (6.5% of all child fall hospitalisations are from high windows; only 10% of such windows have window guards) and low impact (1.5% of all child fall hospitalisations are from high windows; 50% of such windows have window guards). Population figures are specific to children aged 0-14 (2004 data).

### Summary of results: EBD estimates for the WHO European Region

Table 4 combines the high and low scenarios proposed above for lack of window guards with the estimate of the burden of injury associated with lack of smoke detectors to provide ranges of overall death and DALY EBD estimates.

**Table 4:** EBD estimates summary of injury due to housing conditions in the WHO European Region.

high or low	window guards		smoke detectors			
			
impact scenario	deaths children aged <15	DALYs children aged <15 (000s)	deaths all age groups (000s)	DALYs all age groups (000s)	TOTAL deaths (000s)	TOTAL DALYs (000s)
high	18.3	5.70			7.54	203.26
			7.52	197.57		
Low	3.0	0.93			7.53	198.49

In Table 4, injury categories include: child (aged<15) deaths and DALYs from falls from second level or higher windows without window guards; deaths and DALYs due to domestic fires associated with lack of smoke detectors. The estimates are based on WHO Global Burden of Disease data (2004 update - see Tables 5 and 6) and European IDB data.

**Table 5:** DALYs, deaths, population and rates for fire-related injuries in the WHO European Region

Country*	DALYs	deaths	population	DALY rate	death rate
	000s		000s	per million	per million
Albania	1.51	15.4	3,134.4	481.3	4.9

Andorra	0.01	0.2	72.3	78.3	3.2

Armenia	1.76	45.3	3,026.9	582.5	14.9

Austria	0.74	47.1	8,253.4	89.6	5.7

Azerbaijan	10.61	333.3	8,305.9	1,278.0	40.1

Belarus	16.57	729.9	9,847.8	1,682.9	74.1

Belgium	1.86	100.3	10,359.7	179.1	9.7

Bosnia and Herzegovina	0.91	22.1	3,905.3	233.0	5.7

Bulgaria	2.99	109.9	7,794.8	383.6	14.1

Croatia	0.60	46.2	4,539.9	131.4	10.2

Cyprus	0.22	7.4	826.8	265.1	9.0

Czech Republic	1.54	74.4	10,194.5	151.5	7.3

Denmark	0.99	65.9	5,402.9	182.3	12.2

Estonia	2.55	148.8	1,348.3	1,888.2	110.4

Finland	1.66	93.4	5,231.2	316.7	17.9

France	10.25	504.1	60,591.1	169.1	8.3

Georgia	1.29	61.2	4,517.0	284.7	13.6

Germany	8.44	484.0	82,642.6	102.1	5.9

Greece	1.31	103.4	11,079.2	118.5	9.3

Hungary	4.02	178.4	10,113.3	397.5	17.6

Iceland	0.06	2.3	292.9	188.2	8.0

Ireland	0.63	37.2	4,067.7	154.1	9.2

Israel	0.55	19.1	6,574.0	83.3	2.9

Italy	4.68	284.3	58,433.9	80.1	4.9

Kazakhstan	19.10	537.8	15,106.9	1,264.0	35.6

Kyrgyzstan	5.96	83.8	5,152.5	1,155.8	16.3

Latvia	3.74	203.6	2,315.3	1,615.9	87.9

Lithuania	3.35	145.7	3,440.2	974.4	42.4

Luxembourg	0.05	1.7	452.4	114.1	3.8

Malta	0.04	2.1	400.1	91.4	5.3

Monaco	0.00	-	32.4	31.2	-

Netherlands	0.98	43.3	16,263.5	60.4	2.7

Norway	1.14	57.9	4,608.6	247.1	12.6

Poland	18.62	499.7	38,245.5	487.0	13.1

Portugal	1.66	111.4	10,471.6	158.5	10.6

Republic of Moldova	2.94	133.4	3,925.2	748.9	34.0

Romania	11.68	366.8	21,725.8	537.6	16.9

Russian Federation	301.98	12,245.6	144,691.7	2,087.0	84.6

San Marino	0.00	-	29.6	23.2	-

Serbia and Montenegro*	2.58	84.1	10,516.7	245.0	8.0

Slovakia	2.27	55.8	5,386.7	421.6	10.4

Slovenia	0.19	7.5	1,997.2	97.0	3.8

Spain	4.09	220.7	42,778.2	95.7	5.2

Sweden	1.57	113.6	8,997.7	174.0	12.6

Switzerland	0.66	36.0	7,392.1	89.8	4.9

Tajikistan	4.94	102.1	6,467.4	763.8	15.8

The former Yugoslav Republic of Macedonia	0.67	13.1	2,030.3	330.0	6.5

Turkey	32.30	445.0	72,020.5	448.5	6.2

Turkmenistan	13.78	365.9	4,766.0	2,891.7	76.8

Ukraine	60.04	2,736.8	47,247.7	1,270.8	57.9

United Kingdom	7.88	454.0	60,050.6	131.2	7.6

Uzbekistan	26.84	520.4	26,208.8	1,024.2	19.9

**Grand Total**	**604.79**	**23,101.6**	**883,277.0**	**684.7**	**26.15**

Sources:

Population, deaths, DALYs: 2004 from WHO Global Burden of Disease Project, 2004 update http://www.who.int/healthinfo/bod/en/index.html

*Countries are defined as they were in 2004 at the time that these data were collated

**Table 6:** Children aged 0-14: DALYs, deaths, population and rates for child fall-related injuries in European states

Country*	DALYs	deaths	population	DALY rate	death rate
	000s		000s	per million	per million
Albania	3.07	8.4	854.1	3592.1	9.8

Andorra	0.01	0.0	10.4	944.2	1.9

Armenia	1.75	2.7	658.6	2663.0	4.2

Austria	1.32	4.2	1318.4	1003.9	3.2

Azerbaijan	3.39	5.7	2191.1	1547.9	2.6

Belarus	5.54	21.2	1593.8	3475.7	13.3

Belgium	1.96	7.2	1776.5	1101.4	4.1

Bosnia and Herzegovina	1.36	0.0	691.7	1970.8	0.0

Bulgaria	2.59	8.2	1096.8	2361.3	7.5

Croatia	1.07	4.0	716.8	1493.2	5.6

Cyprus	0.18	0.2	168.5	1071.8	0.9

Czech Republic	2.40	3.6	1534.6	1562.8	2.3

Denmark	1.16	0.6	1016.4	1137.0	0.6

Estonia	0.49	0.0	210.6	2326.0	0.0

Finland	1.64	3.0	917.1	1792.9	3.3

France	15.03	35.3	11162.2	1346.3	3.2

Georgia	1.22	6.5	877.8	1384.8	7.5

Germany	9.43	34.6	12066.6	781.7	2.9

Greece	1.54	3.2	1595.0	964.4	2.0

Hungary	2.40	4.5	1618.7	1485.2	2.8

Iceland	0.07	0.3	65.5	1055.1	5.0

Ireland	0.68	2.5	846.1	805.4	3.0

Israel	1.28	2.6	1839.2	697.1	1.4

Italy	8.18	21.8	8202.4	997.8	2.7

Kazakhstan	13.56	75.8	3742.1	3623.4	20.3

Kyrgyzstan	8.25	41.4	1635.8	5043.8	25.3

Latvia	1.07	6.6	346.4	3083.0	19.1

Lithuania	1.50	3.3	597.9	2506.2	5.5

Luxembourg	0.10	0.0	84.3	1214.8	0.0

Malta	0.07	0.3	71.6	999.4	4.8

Monaco	0.01	0.0	6.0	1046.3	4.3

Netherlands	2.24	9.6	3005.0	746.1	3.2

Norway	1.06	0.0	910.0	1163.4	0.0

Poland	20.04	20.5	6434.4	3115.2	3.2

Portugal	2.09	8.5	1647.2	1269.4	5.2

Republic of Moldova	1.95	9.4	816.2	2394.6	11.5

Romania	11.70	31.7	3496.1	3347.1	9.1

Russian Federation	102.32	279.4	22447.1	4558.3	12.4

San Marino	0.00	0.0	4.2	611.9	0.0

Serbia and Montenegro*	3.17	4.4	1976.5	1605.3	2.2

Slovakia	2.56	1.5	930.2	2754.2	1.6

Slovenia	0.42	0.8	287.6	1445.3	2.7

Spain	5.59	22.0	6161.4	907.0	3.6

Sweden	1.33	0.0	1588.0	839.9	0.0

Switzerland	1.16	4.0	1252.2	925.2	3.2

Tajikistan	5.27	36.2	2588.8	2035.8	14.0

The former Yugoslav Republic of Macedonia	0.88	0.0	410.1	2146.2	0.0

Turkey	54.48	162.0	20668.7	2635.8	7.8

Turkmenistan	5.80	26.0	1556.2	3729.9	16.7

Ukraine	21.39	77.4	7155.7	2988.6	10.8

United Kingdom	7.78	14.3	10893.9	713.8	1.3

Uzbekistan	26.65	176.3	8909.1	2991.1	19.8

**Grand Total**	**370.22**	**1191.9**	**162651.6**	**2276.1**	**7.3**

Sources:

Population, deaths, DALYs: 2004 from WHO Global Burden of Disease Project, 2004 update http://www.who.int/healthinfo/bod/en/index.html

*Countries are defined as they were in 2004 at the time that these data were collated

## Discussion

This analysis demonstrated how the use of an EBD approach can be applied to structural injury hazards in the home to estimate the injury burden of these hazards. We showed that two specific hazards, lack of window guards at second floor level and higher, and lack of smoke detectors resulted in an estimated 7,500 deaths and about 200,000 disability adjusted life years (DALYs) per year.

However, the EBD approach has a number of limitations when used to estimate the burden of injury. First, and most importantly, the evidence base for quantifying the exposure-response relationship between housing quality and home injury is small and of poor quality, without control of potential confounders, and based in single countries where country-specific factors may have influenced the outcomes. Second, exposure estimates were rarely available as there are no national surveys of housing quality in Europe outside of England. Some estimates that were not necessarily representative were used in our EBD estimate. For example, to estimate prevalence of housing without smoke detectors, the LARES survey provided single city estimates, which we have used to represent the respective country. For housing characteristics without exposure estimates, we have used a sensitivity analysis derived from using a range of plausible exposures as potential scenarios, tending to be conservative in these choices (by choosing values that were likely to be lower levels or intensities of exposure). A related limitation was a lack of stratification of exposure levels and housing hazards. Housing hazards have strongest effect on injury risk for particular age groups. For example, the lack of fencing of swimming pools presents a higher risk for households with young children. Thirdly, deficiencies in the injury data placed severe limitations on the burden of injury estimation. Injury data need to include information on the setting of the accident to inform injury prevention initiatives and to motivate initiatives via analyses of injury burden such as that in the current paper. Such limitations are likely to be greater for derived measures such as DALYs, which also rely on accurate mortality data.

As the range of housing conditions considered was limited by the exposure data that were available and by gaps in the literature on the exposure-response relationship for many exposures, the burden of injury analysis was restricted to just two injury-hazard combinations. The injury burden associated with housing will be estimated to be considerably larger when limitations can be addressed in the quality of the evidence, the range of exposures measured, and the scope of relevant details of injury circumstances currently recorded in surveillance systems.

England is one of the few countries in the world that carries out a regular survey of private housing quality/conditions, the English House Condition Survey [20]. The 2005/6 survey attempted to capture data on particular serious hazards, defined under a framework developed for the English Housing Health and Safety Rating System, which categorises housing conditions that may increase the likelihood of the occurrence of an adverse health event (such as injury) together with the likelihood of consequent injury or disease of various levels of severity [28]. In the future, it may be possible to use these data to estimate impact fractions for housing injury hazards in England. The data are not available at this stage, however. The United States also carries out a regular representative survey of housing quality (the American Housing Survey) [29], but the data are unlikely to reflect European conditions and are not considered further here.

Limitations in the availability of exposure measures also prevent EBD estimates associated with lack of home air conditioning (leading to heat-related deaths during heat waves). The effectiveness of air conditioning in preventing heat-related mortality has been estimated during a heat wave [14]. As heat waves are unpredictable, there is an added complication in the assessment of this EBD. It should be noted that with climate change there may be higher frequency and severity of heat waves, so domestic air conditioners as well as other housing and environmental features that avoid collecting and trapping heat may play an important role in preserving health, particularly of the very young and very old.

Poisoning is another important injury class that could potentially be reduced by home features such as safe storage cabinets, but no exposure data (prevalence of safe storage in houses), nor evaluations of the efficacy of safe storage exist currently to inform any estimates of poisoning events potentially prevented. The burden of unintentional poisoning is largely imposed on children. In fact for children it is the third leading cause of unintentional injury death: in 2004, 3000 children and adolescents aged 0-19 years died from acute poisoning in the European Region, largely in the home setting [30: page xiii]. Despite these figures, there is reasonably good awareness of prevention methods amongst parents. A recent survey of parents of young children in 14 European countries found that safe storage of poisons/medicines etc. was a commonly cited safety practice amongst these parents [31].

Our estimates of injury burden could be improved in a number of specific ways. (i) Data on housing quality should ideally be available for more countries in the WHO European Region than just England. Such data could inform a considerably more precise estimate of EBD of housing from home injury. (ii) Data for coding the location of the injury need to be improved. The ICD-10 code has a fourth digit assigned to code the location, which includes "home" as one category. Recent analysis has shown that this coding is poorly completed for most European countries [32]. (iii) Similarly, intentional injuries should be analysed separately to unintentional ones. Intentional injuries constitute a large proportion of home injuries [33]. Generally, unintentional injuries are likely to be most amenable to home environmental injury prevention initiatives. The analysis in the current paper was relatively unaffected by the intentionality of the injury. (iv) Researchers should be encouraged to conduct more high quality studies of the relationship between housing quality, home hazards, safety features and home injury. (v) Future EBD analyses could attempt to carry out more sophisticated analyses that consider the distribution of exposures to housing conditions and home injury across different segments of the population and the fact that vulnerability to these exposures varies according to age and other factors. Such analyses should include more injury types and greater ranges of housing conditions.

Despite the limitations listed above, the estimates of home injury burden imply that improving housing quality and the prevalence of safety features such as smoke detectors would reduce home injury levels in Europe, with the consequent reductions dependent on the prevalence of housing hazards. This strategy is therefore likely to be most important in the least developed countries, and the most deprived sub-populations within developed countries, who are likely to suffer from the poorest housing quality. Hazards in the home setting are difficult to study as homes tend to be private spaces, where regulators are reluctant to impose standards or laws, and few surveys are conducted to gather data on injury hazards. This situation leads to a paucity of reliable research to support home injury prevention initiatives and little exposure data on which to build EBD estimates, which are potentially an important motivator for injury prevention policy development and orientation [34].

## Conclusions

The current analysis has identified the fitment of window guards on second floor and higher windows, and the widespread installation of smoke detectors as interventions with some supporting evidence of their effectiveness and a basis on which to estimate exposure levels or exposure scenarios. The instigation of these relatively low-cost measures in the WHO European Region would yield potential savings of about 7,500 deaths and about 200,000 DALYs per year. These measures should be strongly supported by policy initiatives, including regulation wherever possible.

## Abbreviations

DALYs: Disability Adjusted Life Years; WHO: World Health Organization; EBD: Environmental Burden of Disease; IDB: European Injury Database; PAF: Population Attributable Fraction; GBD: Global Burden of Disease.

## Competing interests

The authors declare that they have no competing interests.

## Authors' contributions

MK reviewed the literature, designed the estimation approach, and compiled the injury and exposure data, and drafted the paper. DO identified some exposure data sources. MB designed the framework for the burden of disease report. All authors read and approved the final manuscript.
